# Quality of Life Following Urgent LVAD Implantation for ECMO Therapy in Cardiogenic Shock: A Long-Term Follow-Up

**DOI:** 10.3390/medicina57080747

**Published:** 2021-07-24

**Authors:** Rafal Berger, Hasan Hamdoun, Rodrigo Sandoval Boburg, Medhat Radwan, Metesh Acharya, Reiner Markus Waeschle, Christian Schlensak, Aron-Frederik Popov, Petar Risteski

**Affiliations:** 1 Department of Thoracic and Cardiovascular Surgery, University of Tuebingen, Hoppe-Seyler-Strasse 3, 72076 Tuebingen, Germany; hasan.hamdoun@med.uni-tuebingen.de (H.H.); rodrigo.sandoval-boburg@med.uni-tuebingen.de (R.S.B.); medhat.radwan@med.uni-tuebingen.de (M.R.); christianschlensak@med.uni-tuebingen.de (C.S.); aron-frederik.popov@med.uni-tuebingen.de (A.-F.P.); petar.risteski@med.uni-tuebingen.de (P.R.); 2 Department of Cardiac Surgery, Nottingham City Hospital, Hucknall Rd., Nottingham NG5 1PB, UK; metesh.acharya@doctors.org.uk; 3 Department of Anaesthesiology, University of Goettingen, Robert-Koch-Strasse 40, 37075 Goettingen, Germany; rwaeschle@med.uni-goettingen.de

**Keywords:** LVAD, cardiogenic shock, quality of life, follow-up

## Abstract

*Background and Objectives*: Over the past decade, veno-arterial extracorporeal membrane oxygenation (VA-ECMO) has developed into a mainstream treatment for refractory cardiogenic shock (CS) to maximal conservative management. Successful weaning of VA-ECMO may not be possible, and bridging with further mechanical circulatory support (MCS), such as urgent implantation of a left ventricular assist device (LVAD), may represent the only means to sustain the patient haemodynamically. In the recovery phase, many survivors are not suitably prepared physically or psychologically for the novel issues encountered during daily life with an LVAD. *Materials and Methods*: A retrospective analysis of our institutional database between 2012 and 2019 was performed to identify patients treated with VA-ECMO for CS who underwent urgent LVAD implantation whilst on MCS. Post-cardiotomy cases were excluded. QoL was assessed prospectively during a routine follow-up visit using the EuroQol-5 dimensions-5 level (EQ-5D-5L) and the Patient Health Questionnaire (PHQ-9) surveys. *Results*: Among 126 in-hospital survivors of VA-ECMO therapy due to cardiogenic shock without prior cardiac surgery, 31 (24.6%) urgent LVAD recipients were identified. In 11 (36.7%) cases, cardiopulmonary resuscitation (CPR) was performed (median 10, range 1–60 min) before initiation of VA-ECMO, and in 5 (16.7%) cases, MCS was established under CPR. Mean age at LVAD implantation was 51.7 (+/−14) years and surgery was performed after a mean 12.1 (+/−8) days of VA-ECMO support. During follow-up of 46.9 (+/−25.5) months, there were 10 deaths after 20.4 (+/−12.1) months of LVAD support. Analysis of QoL questionnaires returned a mean EQ-5D-5L score of 66% (+/−21) of societal valuation for Germany and a mean PHQ-9 score of 5.7 (+/−5) corresponding to mild depression severity. When compared with 49 elective LVAD recipients without prior VA-ECMO therapy, there was no significant difference in QoL results. *Conclusions*: Patients requiring urgent LVAD implantation under VA-ECMO support due to CS are associated with comparable quality of life without a significant difference from elective LVAD recipients. Close follow-up is required to oversee patient rehabilitation after successful initial treatment.

## 1. Introduction

Over the past decade, veno-arterial extracorporeal membrane oxygenation (VA-ECMO) has developed into a mainstream treatment for refractory cardiogenic shock (CS) to maximal conservative management [1,2]. Since successful weaning off of VA-ECMO may not always be feasible, bridging with further mechanical circulatory support (MCS) such as an urgent implantation of a left ventricular assist device (LVAD) may remain the only alternative to support the patient haemodynamically [3]. Following VA-ECMO, reported survival to hospital discharge and to one year varies from 30% to 60% due to successful initial treatment [1,2,4,5]. After recovery, new medical concerns and practical challenges faced by survivors when being rehabilitated to daily life with an LVAD may impact significantly on their quality of life (QoL).

Comparison of the QoL of patients who have received an LVAD device on an elective basis with emergent recipients might help to understand how much improvement in QoL can be expected after urgent and unexpected LVAD implantation.

Despite existing knowledge surrounding hospital survival following VA-ECMO therapy, there is a paucity of data on longer-term outcomes and quality of life of VA-ECMO survivors [2,6]. The purpose of our study was to evaluate the long-term effects on QoL in patients undergoing urgent LVAD implantation as part of VA-ECMO therapy for refractory CS, and to determine whether any difference exists when compared with elective LVAD recipients.

## 2. Materials and Methods

### 2.1. Patient Selection

A retrospective analysis of our institutional database was performed to identify all LVAD implantations in adult patients over the 8-year period between 1 January 2012 and 31 December 2019. Each case was analysed in terms of MCS before surgery. Patients who received a replacement of the LVAD or other cardiac circulatory support device were excluded from further analysis after the second operation. Furthermore, urgent LVAD implantations with inotropic support but without MCS before surgery, post-cardiotomy CS and LVAD implantations in paediatric patients were excluded. We finally categorised patients into either a ‘VA-ECMO’ or ‘elective’ group, according to urgency of LVAD implantation.

### 2.2. Quality of Life

Quality of Life questionnaires were conducted prospectively at our centre during elective follow-up visits of survivors of the initial therapy. The cut-off date for analysis was 31 December 2020 so that a minimal observation time of one year for the last implantation was achieved. Study participants were consecutive patients who underwent continuous-flow implantable LVAD therapy, including HeartMate II (Thoratec Corporation, Pleasanton, CA, USA), HeartWare (Medtronic, Dublin, Ireland) and HeartMate III (Thoratec). The QoL questionnaires were performed at 3, 6 and 12 months after implantation. In longer-term survivors, the last available results from over 12 months were additionally included.

Two types of QoL questionnaires were used. The EuroQol-5 dimensions-5 level (EQ-5D-5L) survey is a generic tool to measure health-related QoL and consists of 5 dimensions: mobility, self-care, usual activities, pain/discomfort and anxiety/depression [7]. Each dimension has 5 levels of severity: no problems, slight problems, moderate problems, severe problems and extreme problems. The total score is evaluated as a percentage, with higher values indicating a better QoL [7,8]. The Patient Health Questionnaire (PHQ-9) is a 9-question depression scale used to predict the presence and severity of depression. Responses range from ‘0’ to ‘3’ and the total score ranges from 0 to 27 with higher scores indicating a greater severity of depression. Values 0–4 represent a minimal depression, 5–9 a mild depression and >10 a moderate depression with need for treatment [9].

### 2.3. Statistical Analysis

Categorical data are presented as frequencies and percentages. For continuous variables, mean and standard deviations (SD) or median and interquartile range (IQR) are reported. In order to compare characteristics among two groups of patients, we performed a Mann–Whitney U test for non-parametric variables and Student’s *t*-test for parametric variables. Normality was tested with the Kolmogorov–Smirnov and Shapiro–Wilk tests. The chi-square test was applied to test relationships between the categorical variables. A statistical significance was defined as *p* < 0.05. All analyses were performed using the SPSS 26 (IBM Corporation, Armonk, NY, USA) software.

### 2.4. Ethical Approval

Ethical approval for this study was sought from the Ethics Committee Board at our institution (Ref. 194/2020BO2). The need for written consent was waived.

## 3. Results

**Figure 1:**
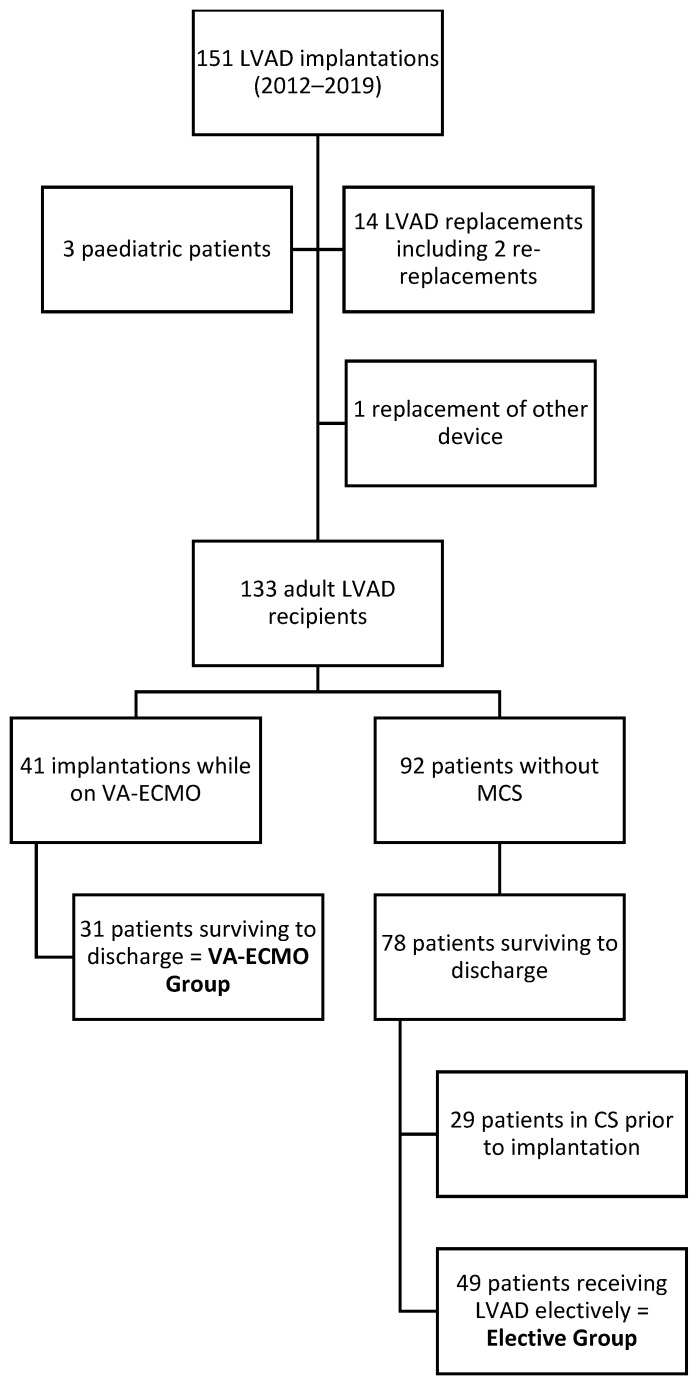
Flow chart of study population selection.

A total of 133 adult patients underwent a total of 147 LVAD implantations (Figure 1), including 13 LVAD replacements and one replacement of another implanted cardiac circulatory support device in our centre during the study period. A total of 41 (30.8%) patients received an LVAD while on VA-ECMO, with 10 post-operative in-hospital deaths, resulting in 24.4% mortality. In total, 109 (82%) patients were alive at discharge. In this population, there were 31 (28.4%) cases of urgent LVAD implantation performed as a bridging from VA-ECMO, and this cohort comprised the ‘VA-ECMO’ group. Among the remaining 79 (72.5%) survivors, 30 cases were excluded due to pre-operative inotropic support before LVAD implantation. Finally, a subgroup of 49 (45%) survivors was included in the ‘elective’ group.

Study participants’ demographic and clinical characteristics are described in Table 1. The VA-ECMO population was significantly younger and had a longer ICU and overall hospital length of stay. There were no significant differences in sex, aetiology of cardiac disease, left ventricular ejection fraction and follow-up and survival duration. Follow-up was complete in 91.3% (*n* = 73) of cases with 36 (45%) deaths and 44 (55%) patients still alive on the cut-off date (Table 2). Among the seven patients lost to follow-up, four underwent LVAD explantation and heart transplantation was performed in three. The mean follow-up duration was 44.4 (+/−25.7) months with 36 (45%) deaths after a mean interval of 26.7 (+/−17.2) months.

**Figure 2:**
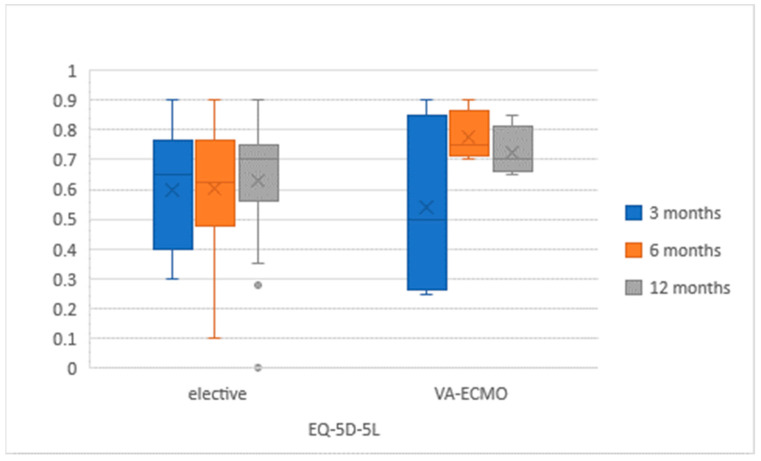
EuroQol-5 dimensions-5 level (EQ-5D-5L) results in study population at 3, 6 and 12 months after LVAD implantation.

**Figure 3:**
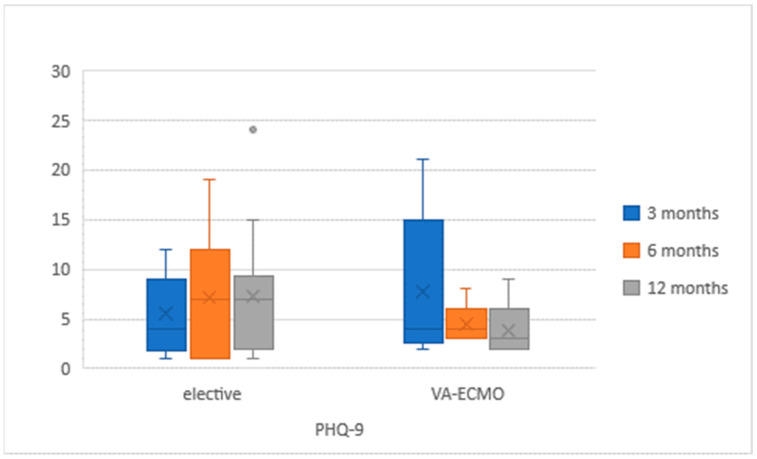
Patient Health Questionnaire (PHQ)-9 results in study population at 3, 6 and 12 months after LVAD implantation.

QoL questionnaires were correctly completed by 39 (48.8%) participants (Figure 2 and Figure 3). The questionnaires were completed by a group of 18 (58.1%) VA-ECMO patients and by 21 (42.9%) patients. Both cohorts did not differ significantly, and the characteristics were similar to these mentioned in Table 1 in terms of demographics and aetiology. In the VA-ECMO cohort, the initial EQ-5D-5L score had improved from 59% (+/−23) after 3 months to 66% (+/−12) after 12 months. In the elective cohort, a similar increase from 58% (+/−12) to 62% (+/−21) was observed. The PHQ-9 score in the VA-ECMO population decreased from an initial 7.2 (+/−6.8) to 5.8 (+/−5.1). In the elective population, the same score increased from 5.8 (+/−4) after 3 months to 7.2 (+/−6.2) after 12 months. There were no significant differences in QoL between both groups of patients at any time of the study (Table 2).

## 4. Discussion

According to recent data, 17% of LVAD implantations are performed in patients in critical cardiogenic shock (INTERMACS 1 and 2) and 17% in patients without any inotropic support (INTERMACS 4 and above) [10]. The outcomes and QoL of VA-ECMO survivors have previously been analysed by other authors, demonstrating LVAD implantation as an effective therapy in patients unable to be weaned from ECMO. Health-related QoL was proven to improve in 3 months after implantation and remained unchanged during 1 year of observation [11]. Nevertheless, a diminished QoL has been found in patients bridged with LVAD, with poorer QoL outcomes observed in patients treated with heart transplantation or from the general population who receive an LVAD [12,13,14]. Nevertheless, there are no studies comparing the patients bridged with LVAD from ECMO with elective LVAD recipients. The fact of being dependent on an assist device is a significant factor that is influencing the QoL regardless of the background.

The purpose of our study was to analyse QoL in LVAD recipients who were bridged from VA-ECMO as a therapy for CS, in comparison with elective implantations. The decision to exclude patients without VA-ECMO support, but with an urgent LVAD indication while on inotropic support from further analysis, is justified, as this cohort was mixed with INTERMACS 2–3 patients who lacked an appropriate period of preparation prior to LVAD implantation. The ‘elective’ group of LVAD recipients included 49 individuals who received the device after a meticulous preparation phase including psychological evaluation as standard at our institution. In all but one case of subacute myocarditis, patients presented with chronic cardiac disease with continuously declining cardiac function, which complicated with over two cardiac decompensations before admission for LVAD implantation (INTERMACS 4–7). Elective patients, however, were provided adequate opportunity to carefully consider the pros and cons of living with a LVAD and were also comprehensively appraised regarding the possible alternative management options. These elective LVAD candidates are thus extremely well-prepared ahead of initiation of costly LVAD support.

In contrast, the 31 patients representing the ‘urgent’ population were in INTERMACS 1 and 2 classes. They were not afforded sufficient preparation time prior to LVAD implantation, owing to the often sudden onset of newly-manifested disease, or their sudden deterioration from an exacerbation of a chronic stable illness. Acute myocardial infarction was the leading cause (41.9%) of VA-ECMO support, followed by arrhythmia (25.8%). LVAD implantation was the only possible treatment modality, with terminal weaning of VA-ECMO as the alternative. The critical clinical status of these patients precluded adequate opportunity for mental preparation and in-depth, time-consuming consideration of all the possible risks of LVAD, such as bleeding, stroke, right heart failure and limitations in daily life (dependence on power supply, bathing, etc.). In every case of acute VA-ECMO implantation, the neurological status of the patient was carefully evaluated beforehand. In cases of CPR preceding VA-ECMO support, we aimed to evaluate patients for LVAD suitability while extubated. Importantly, LVAD implantation was never performed against the will of the patient or the first-degree relatives.

Both groups of patients represent individuals with a contrasting medical history of chronic cardiac disease versus cardiogenic shock with the need for cardiopulmonary resuscitation, the latter in 35.5% of cases. Nevertheless, in our prospective study with extended follow-up duration, we did not identify any significant difference in the QoL of LVAD recipients, whether elective patients or survivors bridged from VA-ECMO (Table 2). Both urgent and elective LVAD recipients could achieve a long follow-up without an influence on duration and mortality. According to patient-completed questionnaires, an acceptably good score of EQ-5D-5L questionnaire and a moderately low PHQ-9 score corresponding to a mild depression were noted in long-term survivors [7,9].

The literature reports a 1-year survival rate for patients after LVAD implantation bridged from VA-ECMO, ranging between 50% and 78% [2,13,15]. In our group of 41 implantations and 31 survivors, there were three deaths during one year of follow-up accounting for a 75.6% in-hospital, and 68.3% 1-year, survival. After 46.9 (+/−25.5) months of observation, 21 (51.2%) survivors of VA-ECMO therapy were still alive while on LVAD support.

In a previous analysis, Lamba et al. compared the outcomes of 26 LVAD recipients who were bridged with VA-ECMO with 107 LVAD recipients without previous MCS. The authors did not find a significant difference in 30-day mortality and 1-year survival between either group (53.8% in VA-ECMO and 60.9% in non-ECMO group) [16]. Our study reiterates these findings whilst also establishing that the QoL of VA-ECMO survivors is non-inferior to that of elective LVAD patients.

Orthotopic heart transplantation may be considered in patients not amendable to weaning from VA-ECMO, although donor availability remains a significant limiting factor. In a meta-analysis by Huckaby et al. considering follow-up in 13,142 patients following heart transplantation, there were no significant differences in the 90-day Karnofsky score regardless of bridging modality, including inotropic support, intra-aortic balloon pump, VA-ECMO and no support before surgery [17]. In an analysis by DeFilippis et al. of 906 patients receiving ECMO, there was no difference in mortality between 587 patients bridged to LVAD and 319 patients receiving a donor heart. The authors concluded that bridging with LVAD was non-inferior to transplantation in VA-ECMO patients [18].

Our results are limited to the experience of one centre and to patients treated with VA-ECMO for a CS with the exclusion of post-cardiotomy cases. Kowalewski et al. showed recently in their meta-analysis that the outcomes of VA-ECMO therapy vary significantly between institutions with heart-transplant or VAD programmes, and without such bridging options, with in-hospital mortality of 55.8% and 65.5%, respectively [19]. Such factors have, without a doubt, a significant influence on the QoL of survivors of VA-ECMO after initial success and following hospital discharge. Furthermore, we did not evaluate the original selection process for patients who were bridged with LVAD, as there were no specific criteria for this. Decision making for weaning, bridging and termination of VA-ECMO therapy was a multi-disciplinary process, and inclusive of the patient’s and relatives’ wishes. The QoL analysis was performed after hospital discharge and results were not compared with preoperative status and included only survivors. Finally, the QoL questionnaires were completed solely by the patients without independent evaluation, and thus represent subjective assessment.

## 5. Conclusions

Whilst VA-ECMO is a high-risk initial treatment in CS, satisfactory long-term outcomes can be achieved in patients bridged with LVAD, where VA-ECMO cannot be weaned. Survival and QoL of VA-ECMO survivors do not differ significantly from that of elective LVAD recipients. In our opinion, LVAD implantation is an acceptable alternative and should be considered as an additional management option in VA-ECMO patients.

## Figures and Tables

**Table 1:** Characteristics of study population in 2 groups.

	All (*n* = 80)	VA-ECMO (*n* = 31)	Elective (*n* = 49)	*p* Value
Demographics:				
Age (years, SD)	55.5 (+/−13.3)	51.7 (+/−14)	57.9 (+/−12.4)	**<0.05**
Sex (male, %)	69 (86)	25 (80.6)	44 (89.8)	0.25
Aetiology:				
Ischemic cardiomyopathy (*n*, %)	44 (55)	17 (54.8)	27 (55.1)	0.515
Dilated cardiomyopathy (*n*, %)	31 (38.8)	10 (32.3)	21 (42.9)	0.56
Myocarditis (*n*, %)	4 (5)	3 (9.7)	1 (2)	0.129
Post-partum cardiomyopathy (*n*, %)	1 (1.3)	1 (3.2)	-	
Pre-operative status:				
LVEF (%, SD)	16.7 (+/−6.1)	15.1 (+/−6.8)	17.5 (+/−5.6)	0.082
Acute infarction (*n*, %)		13 (41.9)	N/A	
Arrhythmia (*n*, %)		8 (25.8)	N/A	
Out of centre VA-ECMO (*n*, %)		10 (32.3)	N/A	
CPR prior to implantation (*n*, %)		11 (35.5)	N/A	
CPR duration (min., range)		14.9 (1–60)	N/A	
Implantation under CPR (*n*, %)		5 (16.1)	N/A	
Duration of VA-ECMO support (days, SD)		12.1 (+/−8)	N/A	
Outcome:				
ICU stay (days, SD)	21 (1–97)	36 (11–97)	12 (1–55)	**<0.05**
Hospital stay (days, range)	42 (11–208)	59 (18–208)	32 (11–100)	**<0.05**
Follow-up (months, SD)	44.4 (+/−25.7)	46.9 (+/−25.5)	42.1 (+/−26.2)	0.526
Mortality (*n*, %)	36 (45)	10 (32.3)	26 (53.1)	0.07
Survival-to-death (months, SD)	26.7 (+/−17.2)	20.4 (+/−12.1)	29.2 (+/−18.5)	0.244

CPR, cardiopulmonary resuscitation; ICU, intensive care unit; LVEF, left ventricular ejection fraction; SD, standard deviations; VA-ECMO, veno-arterial extra-corporeal membrane oxygenation, *p* Value significant if below 0.05.

**Table 2:** Quality of life of study participants.

	VA-ECMO (*n* = 31)	Elective (*n* = 49)	*p* Value
EQ-5D-5L			
3 months	59% (+/−23)	58% (+/−18)	0.848
6 months	72% (+/−16)	62% (+/−21)	0.233
12 months	66% (+/−15)	62% (+/−21)	0.866
Last available *	66% (+/−21)	65% (+/−23)	0.944
PHQ-9			
3 months	7.6 (+/−6.8)	5.8 (+/−4)	0.49
6 months	5.3 (+/−3.5)	6.4 (+/−5.1)	0.733
12 months	5.8 (+/−5.1)	7.2 (+/−6.2)	0.614
Last available *	5.7 (+/−5)	5.6 (0–22)	0.776
	* after 46.9 (+/−25.5) months	* after 42.1 (+/−26.2) months	

EQ-5D-5L, EuroQol-5 dimensions-5 level; PHQ-9, Patient Health Questionnaire; VA-ECMO, veno-arterial extra-corporeal membrane oxygenation. * Refers to last available data, which differs in both groups.

## Data Availability

No additional data included.
